# Periodic 48 h feed withdrawal improves glucose tolerance in growing pigs by enhancing adipogenesis and lipogenesis

**DOI:** 10.1186/1743-7075-9-10

**Published:** 2012-02-09

**Authors:** Priya S Mir, Mao L He, Gregory Travis, Toby Entz, Tim McAllister, Sigrid Marchand, Al Schaefer, Jon Meadus, Pierre Lepage, Erasmus Okine, Michael V Dodson

**Affiliations:** 1 Agriculture and Agri-food Canada Research Centre, 5403, 1st Ave S., P.O. Box 3000, Lethbridge, AB T1J 4B1, Canada; 2 Agriculture and Agri-food Canada Research Centre, 6000 C and E Trail, Lacombe, AB T4L 1W1 Canada; 3 Department of Agriculture, Food and Nutritional Science, University of Alberta, Edmonton, AB T6G 2H1, Canada; 4 Department of Animal Sciences, Washington State University, Pullman, WA 99163-646351 USA

**Keywords:** Adipocyte diameter, area under the curve, glucose tolerance, glucose incorporation into fat, pigs, feed withdrawal

## Abstract

**Background:**

Adipocyte numbers and peroxisome proliferators activated receptorγ (PPARγ) expression of retroperitoneal tissue increased while area under the curve (AUC) during the glucose tolerance test (GTT) was reduced in rats subjected to certain feed withdrawal (FW) regimens. Thus, using pigs as the experimental model, the hypothesis that FW regimens influence glucose tolerance by influencing fat cell function was evaluated with the objective of determining the effect of a single (FWx1; at age of 19 wk for 48 h) or periodic, multiple (FWx4; 24 h FW at 7 and 11 wk of age and 48 h FW at 15 and 19 wk of age) FW on AUC of glucose and insulin during the GTT relative to pigs that did not experience FW (Control).

**Methods:**

Growth, body composition, adipocyte numbers, PPARγ expression, lipogenic potential as glucose uptake into fat of adipocytes of varying diameter in omental (OM) and subcutaneous (SQ) fat as affected by FW regimens were determined in pigs initiated into the study at 5 wk of age and fed the same diet, ad libitum.

**Results:**

Blood glucose concentrations for prior to and 120 min post glucose meal tended to be lower (p = 0.105 and 0.097, respectively) in pigs in FW treatments. In OM fat; cell numbers, glucose Universal^14^C [U^14^C] incorporation into fat and rate of incorporation per 10^4 ^cells was greatest for cells with diameters of 90-119 μm. Pigs undergoing FWx4 tended to have greater (p = 0.0685; by 191%) number of adipocytes, increased (p = 0.0234) glucose U^14^C incorporation into adipocytes and greater (p = 0.0872) rate of glucose uptake into cells of 119-150 μm diameter than of cells from control or FWx1 pigs. Subcutaneous adipocyte numbers in 22-60 and 61-90 μm diameter ranges from pigs in FWx1 tended to be greater (p = 0.08 and 0.06, respectively) than for those in FWx4 treatment, yet PPARγ expression and total cell number were not affected by treatment.

**Conclusions:**

Results suggest that FW regimens influence fat cell function or lipogenesis rather than number, affecting glucose metabolism and may have implications in drug-free control of metabolic syndrome symptoms.

## Background

The increase in incidence of clinical disorders that arise from metabolic syndrome has led to substantial research investment in this area, resulting in significant developments in the understanding of the progression of the syndrome. The manifestation of metabolic syndrome symptoms may not be related to obesity alone [1], but could be associated with the ability of adipose tissue to synthesize fat or be functional in maintenance of energetic homeostasis in the body and operate as a buffer between consumed energy and that utilized for life activities [2]. Contrary to earlier observations [3] it is now hypothesized that lipid-saturated adipocytes, of relatively larger diameter (greater than 135 μm), have compromised lipogenic potential with poor buffering action leading to increased blood lipids, and perhaps elevated plasma glucose. These problems may lead to ectopic lipid deposition along with manifestation of adipose tissue inflammation resulting in insulin resistance [4]. Protection against inflammation, thus insulin resistance in humans appears to reside in the enzymatic ability of adipocytes to synthesize fat [5]. Inability to synthesize and store fat, as in lipodystrophic non-obese humans and mice, led to development of insulin resistance [6], while adipose tissue transplants into these mice alleviated symptoms of hyperglycemia, hyperinsulinemia and dyslipidemia [7,8], suggesting that the retention of the ability to enhance adipocyte differentiation or lipogenic potential can be protective against insulin resistance. Similarly, decline in insulin resistance in thiazolidinedione treated type-2 diabetics appears to be mediated via increase in adipocyte differentiation [9]. The preceding review suggests that protocols that increase adipocyte numbers with lipogenic capacity could abate progression of metabolic syndrome in humans by increasing the energy buffering or functional capacities of adipocytes.

Adipocyte numbers tended to increase in intramuscular and subcutaneous adipose tissues of beef cattle subjected to a single feed withdrawal (FW) for 48 h prior to initiation of the fattening phase [10] with positive economic returns due to elevated quality grades. Although it has been known that feed restriction (provision of energy deficient diets) followed by ad libitum feeding enhances production efficiency of livestock [11], the effect of FW for 48 h prior to start of the fattening phase on adipocyte number in intramuscular fat was first addressed by Mir et al. [10]. While effects of fasting have been studied in the rat, as models for humans [12,13], the focus has been on lipoprotein lipase activity. However, He et al. [14] reported that fa/fa obese rats; that underwent fortnightly feed withdrawal for 24 h, had increased expression of the adipocyte marker; peroxisome proliferator-activated receptorγ (PPARγ) along with a 13% increase in total cell density in the retroperitoneal adipose tissue. He et al. [14] reported a significant, quadratic relationship between average cell diameter of the retroperitoneal tissue and the area under the curve (AUC) for glucose during a glucose tolerance test (GTT) in Wistar male rats. As a result of these observations the authors suggested that glucose management relied on fat synthetic activity of adipocytes, which was a function of both; adipocyte density and size. To date no investigation has addressed the effect of FW regimens, on adipocyte characteristics and consequently on symptoms of metabolic syndrome in pigs even though pigs could be ideal as models for humans for the study of metabolic syndrome [15]. The present study was conducted to determine whether FW regimens would alter adipocyte number and size (diameter) in the omental (OM) and subcutaneous (SQ) adipose tissues, and if these factors influenced AUC of glucose and insulin during a GTT. Furthermore, it was crucial to determine if FW regimens affected glucose incorporation into fat [4] in OM and SQ adipose tissues in cells that were divided into five diameter ranges from 22 to 288 μm [14,16] rather than three ranges from 20 to 150 μm [4] and whether cell number and cell diameter were affected. The effect of FW regimen on PPARγ expression in the adipose tissues was necessary because it is recognized as an index of an increase of insulin sensitive adipocytes, which appear to control progression of metabolic syndrome [17]. Thus the hypothesis that reductions of AUC for glucose during a GTT could be achieved through FW regimen because of an effect on lipogenic potential was evaluated. The objectives of the present investigation, were to determine the effects of a single FW (FWx1) for 48 h at 19 wk of age or multiple FW (FWx4) for 24 h at seven and 11 wk and for 48 h at 15 and 19 wk of age in castrated pigs between the ages of 5 and 23 wk as models for humans on growth of pigs, body composition, OM and SQ adipose tissue cellularity by adipocyte diameter (μm), glucose incorporation into fat and AUC for glucose and insulin.

## Methods

### Animals and treatments

An experiment was conducted with 27, weanling, castrated, 5 wk old pigs after obtaining the approval of Lacombe Research Centre Animal Care Committee (approval number: 20901) of Agriculture and Agri-Food Canada. The pigs were cared for according to the guidelines of the Canadian Council on Animal Care [18]. The 27 pigs were individually housed in pens with slatted floors in rooms maintained on a 12 h light:dark cycle at 18°C. The pigs in all FW treatments were provided with the same diet and were exposed to pre-grower, grower and finisher rations produced by Wetaskiwin Co-op feeds, Wetaskiwin, Alberta to meet or exceed recommendations detailed by the National Research Council [19]. Feed protein concentrations ranged between 23.7 and 13.2% based on weight of the pigs and the digestible energy available from the diet was 340 Kcal/100 g of diet derived from cereals and protein supplements.

**Figure 1:**
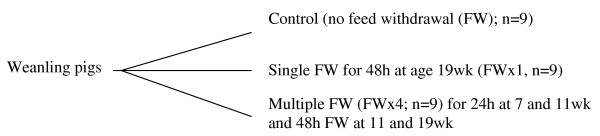
**Schematic for application of feed withdrawal (FW) treatments**.

The castrated pigs were progeny of Large White X Landrace crossbred females that were bred to Durok boars, and were obtained from Hypor Comp (Alberta). The pigs were provided feed and water ad libitum and allowed to acclimatize to the barn for 2 wk. The following day, pigs were weighed, randomized by weight to the three treatments at nine pigs per treatment as a completely randomized design experiment. The treatments assigned to the weanling pigs were as described in the schematic in Figure 1.

Post acclimatization, at initiation of the experiment, the pigs were weighed and placed in pens with cleaned feed bunks and those in the FWx4 treatment were not provided feed. After 24 h the feed was provided to the pigs in treatment FWx4. The pigs in the no FW (control) and FWx1 treatments were provided feed ad libitum post weighing. All pigs had free access to water at all times. The pigs were weighed every 4 wk. After the weight was recorded, the pigs in the FWx4 treatment were denied feed for 24 h at 11 wk and for 48 h at 15 and 19 wk of age. The pigs in the FWx1 treatment were denied feed for 48 h post weighing at the age of 19 wk. The pigs were maintained in experiment for 18 wk or until the age of 23 wk. The GTT was conducted during the 16^th ^wk of the experiment, at the rate of three pigs each day, in nine pigs with three pigs randomly selected from each treatment. At 23 wk of age or after 18 wk in experiment the pigs were euthanized in a federally inspected abattoir (3 pigs/d/treatment within the wk) and samples of OM and SQ adipose tissue and of the longissimus muscle were procured within 15 min of exsanguination for determination of glucose Universal ^14^C [U ^14^C] incorporation into fat and adipocyte enumeration and molecular marker PPARγ in the tissues.

### Glucose tolerance test (sub-experiment 1)

The GTT was conducted in nine pigs when they were of 21 wk old or during the 16^th ^wk of the experiment. Pigs were catheterized for ease of blood sampling one day prior to the test. An indwelling ear vein catheter (Intramedic tubing, PE 90, 0.86 mm and 1.17 mm internal and outer diameter, respectively; Becton Dickinson; Parsippany, N.J.) was inserted under aseptic conditions approximately 20 cm intravascularly. A pig from each treatment was fasted for 24 h and the blood was collected to obtain fasting blood for glucose and insulin determination, after which the glucose was provided in a small amount of feed and the time for complete consumption of the feed was monitored, which was always less than 15 min. Glucose was provided at 1.4 g/kg bodyweight. Blood samples were collected for determination of glucose and insulin concentration in the blood and plasma, respectively. Blood was drawn prior to and after 5, 15, 30, 45, 60, 90, 120, and 150 min post ingestion of the glucose meal. Blood glucose was determined using a hand held glucometer (MediSense, Abbot Laboratories, Montreal, QC., Canada) and Precision Xtra blood glucose strips (MediSense, Abbot Laboratories, Montreal, QC., Canada) [20,21] prior to and post consumption of the glucose meal. The glucose concentration was plotted against time and the area under the curve (AUC) was calculated as AUC_Ground _(AUC_G _mmol/L * min; [22]). At the times of blood collection for blood glucose measurement, samples were obtained for determination of plasma insulin concentration. Approximately 10 mL of blood was collected in evacuated tubes (Beckton and Dickenson, Mississauga, ON, Canada) containing potassium EDTA and placed on ice. The blood was centrifuged at 600 × g for 10 min at 5°C and the plasma was separated and frozen until the samples were analyzed for insulin concentration. Insulin concentration in plasma was determined using the porcine insulin kit (Bio Vendor with catalogue #RSHAKRIN013TR for Shibayagi Co. Ltd., Gunma Japan ELISA kit) and the CV for the control used in the estimation was 8.28. The AUC_G _for insulin was determined as for glucose [22]. The fasting glucose and insulin concentrations were used to calculate the homeostatic model assessment (HOMA) index for the pigs [23].

### Cellularity and glucose incorporation into fat

At euthanasia tissue samples were collected from the OM and the SQ and glucose incorporation into fat was evaluated by measuring uptake of glucose universal ^14^C [U ^14^C] as counts per minute (CPM) into the fat extracted from the cells from each tissue [4]. Briefly each tissue sample from each animal was collected and placed in Tris sucrose buffer at 37°C (30 mM Tris-HC1, 0.3 M sucrose, 1 mM EDTA, 1 mM glutathione; pH **7.4 **at 37°C) and transported to the radioactive laboratory for incubation. Samples of adipose tissue of 200-300 μm in thickness were cut, rinsed twice with buffer without EDTA (30 mM Tris-HC1, 0.3 M sucrose, 1 mM glutathione; pH 7.4 at 37°C) and approximately 100-150 mg of tissue was placed in 25 mL test tubes for subsequent incubation.

Duplicate adipose tissue slices were incubated in 25 mL test tubes which contained 3 mL of Krebs-Ringer bicarbonate (KRB) buffer, pH 7.4, with half the calcium and 30 mg/mL of fat free, de-colourized bovine serum albumin. The KRB buffer also contained 10 μM of glucose (Vmax for substrate concentration) and 0.3 μCi/mL of glucose U ^14^C (New England Nuclear Waltham Massachusetts, USA). The test tubes were flushed with O_2_:CO_2_, (95:5), sealed with a rubber serum cap, and incubated at 37°C for 2 h in a shaking water bath. Following incubation, tissue slices were rinsed three times in 37°C, 0.154 M NaCl, and once in 3 mL of 50 mM collidine-HCl buffer, pH 7.4, blotted carefully, and weighed into vials containing 2 mL of 3% osmium tetroxide in collidine buffer for fixation of cells [24] and held at room temperature with occasional shaking for 96 h to ensure that all the tissue was fixed. After the 96 h, the osmium tetroxide was removed and 10 mL of 0.154 M NaCl was added and held at room temperature for 24 h after which the 0.154 M NaCl was removed and 10 mL of 8 M urea in 0.154 M NaCl was added and the tissue allowed to soften for 48 h or until cells were liberated from the tissue matrix. The urea was removed and 10 mL of distilled water was added and the cells were transferred to 50 mL tubes followed by 3 washes of, 5 mL aliquots of 0.154 M NaCl, pH 10 to rinse the cells completely into the tubes. Fixed adipocytes were separated into five sizes based on diameter to determine cellularity and measure glucose incorporation as CPM in fat extracted from the cells separated by diameter size.

### Cell fractionation into five diameter ranges

Nitex material (Sefar Canada, St. Laurent, Qc) with openings of 280, 150, 118, 90, and 60 μm were used to successively filter the cells released from the fixed fat tissue samples and cells remaining over the nitex post filtration was collected with each of 3 washes of 2 mL 0.154 M NaCl. The filtration process divided the cells into fractions of cells in diameter ranges of 151-288, 119-150, 91-118, 61-90 and less than 60 μm. The cell material remaining on the 288 μm nitex was discarded. Cell numbers in each fraction were determined by computer image analysis (Motic Images Plus 2.0 ML, Motic China Group Co. Ltd., Xiamen, China) [14] of 1 mL of the adipocyte suspension in a Sedgwick-Rafter counting chamber (Thomas Scientific, Swedesboro, NJ) [25]. The adipocyte suspension was prepared by adding an equal volume of glycerol to each cell suspension and further dilutions if necessary were prepared by adding known and required volumes of 40% glycerol in 0.154 M NaCl. All the dilution factors applied to each cell suspension were recorded and used in determination of cell number within each diameter range for each sample.

### Measurement of radioactivity

The cell fractions by diameter range collected from filtration were drained free of the glycerol-saline and rinsed with distilled water to remove the glycerol and the suspension was rinsed into a glass scintillation vial with water. The water was evaporated in a heating block (Pierce Reacti-Therm III Heating Module; Rockford, IL, 61105) at 60°C until approximately 1 mL remained. In order to remove the Osmium tetroxide 3 mL of hydrogen peroxide (30%) was added to each cell suspension and heated in the block heater (in a ventilated fume hood) to volatilize Osmium tetroxide from the adipocytes. This was repeated to ensure total loss of color followed by evaporation of water until approximately 1 mL remained. The samples were cooled and the fat in the decolorized cells was extracted by vortexing with 2 mL of chloroform and 1 mL of methanol for 30 s. The samples were centrifuged and the chloroform layer was collected into scintillation vials. The extraction was repeated to ensure complete extraction of the fat in the cells. The chloroform was evaporated under a stream of nitrogen then 4 mL of Ultra-Gold scintillation cocktail was added, vortexed at high speed for 30 s and the Beta counts were measured using the Beckman Liquid Scintillation System LS 6000 IC (Beckman Inst. Inc. Fullerton, CA) and the CPM in fat was calculated for cells in each diameter range per mg of sample fixed and per 10^4 ^cells. In order to determine the glucose incorporation into fat, the average of the blank CPM, collected three times during each day of measurement (3d) were first deducted from the CPM of the fat extracted from the cells, which represented incorporation of D- glucose [U ^14^C] into total lipid and the radioactivity was measured for each cell fraction from the cells from the original weight of the tissue incubated.

### RNA isolation

Omental fat was collected from the abdominal region of adult swine at the time of euthanasia in RNase later (Ambion) and frozen at -80°C. RNA was isolated from the fat using the Aurum total RNA fatty and fibrous tissue kit (Biorad). The kit includes a preliminary DNase clean up step to insure no genomic DNA contamination.

The RNA samples were quantified spectrophotometrically at 260 nm. All RNA isolates had an OD_260_:OD_280 _between 1.8 and 2.0, indicating clean RNA isolates. The RNA quality was also checked for the presence of 28S and 18S rRNA band on 1.0% agarose gel electrophoresis stained with 1 μg/mL ethidium bromide.

### Relative reverse transcriptase - polymerase chain reaction (RT-PCR) analysis

A two step quantitative reverse transcriptase (RT-PCR) method was used to measure gene expression in the samples. In the first step, cDNA synthesis was made from total RNA (5 ug) with 0.5 μM oligo-dT, 0.2 μM random hexamer primers, 500 μM of each dNTPs and H_2_0 and preheated at 65°C for 2 min to denature secondary structures. The mixture was then cooled rapidly to 20°C, then 10 μL 5× RT Buffer, 1 unit/μl of Rnase inhibitor and 200 U MMLV Reverse Transcriptase (Sigma-Aldrich, Oakville, ON, Canada) was added for a total volume of 50 μL. The RT mix was incubated at 37°C for 90 min. then stopped by heating at 95°C for 5 min. The cDNA stock was stored at -20°C. The yield of cDNA was measured according to the glycerol-3-phosphate dehydrogenase (GADPH) [SSU97256] 5'-atctgtgaccagctcaagg-3' and 5'-gcaggaggtggacacagt-3' and beta-actin [AY550069] 5'-acatcaaggagaagctgtgc-3' and 5'-gcaaggacctctacgccaa-3' polymerase chain reaction (PCR) signal, generated on a Mx3000 real time PCR machine (Strategene) with SYBR dye detection. Good amplification was determined to be detection in 18 to 22 cycles, starting with 0.1 μL of the cDNA solution in 20 μl of the Quantiech SYBR green kit with 5 pmole of forward and reverse primers of either the GADPH or beta-actin. PCR products were examined after real-time detection by electrophoresis for 2 h at 70 v on native 10% polyacrylamide gels stained with 1 μg/mL ethidium bromide. Genomic DNA contamination was indicated by a 354 bp band and cDNA was indicated by a 256 bp band in the beta-actin amplification. Gene expression of the PPARγ was measured by real-time PCR in a SYBR green reactions using the forward 5'- ataaagtccttccgctgac-3' and reverse 5'-gtatgccaagaacatccctg-3' primers. Gene expression was calculated using GADPH as the reference point, to standardize the data according formula Ct (sample) - Ct (reference) and reported as percent of control.

### Growth hormone (sub-experiment 2)

At time of euthanasia of the pigs, blood was collected into K-EDTA containing tubes from four pigs in the control treatment and another set of four pigs that were not part of the experiment (spares) but had been subjected to FW for 48 h prior to euthanasia and were similarly processed. The plasma was harvested from the blood and analyzed for porcine growth hormone (Cedarlane product #E0044PO from USCN Life Science Inc, Wuhan China). The CVs for the growth hormone values ranged from 0.9 to 16.0 among the animals that underwent FW and from 6.2 to 12.5 for the samples from the animals in the control treatment.

### Statistical analysis

The data from the experiment were analyzed as a completely randomized design experiment [26] using PROC MIXED of SAS (SAS Inst., Cary, NC, 2005). Data from the pigs were analyzed for variance and mean separation using Least Square Difference [26] was applied only if the *F*-value was significant for treatments. The experimental unit was the pig because they were individually penned and all data were collected for each pig. Correlation coefficients between AUC for glucose and insulin from the GTT versus glucose and insulin concentrations at the times of sampling were determined. For the glucose [U ^14^C] uptake study only values from pigs where the sample size incubated was less than the 160 mg was included in the data analysis, thus the replication for this aspect of the study for the control, FWx1 and FWx4 treatments was five, six and seven, respectively for the OM tissue and seven, six and seven for SQ tissue respectively. Samples incubated for glucose uptake from most (six out of nine) of the pigs that were used for the GTT was larger than the 160 mg as a result these were not included and cellular effects on GTT parameters could not be studied. Values are expressed as mean ± SEM and differences were considered significant at p < 0.05, while values between p > 0.05 < 0.1 were considered as expressing a trend. Difference between average target mRNA for PPARγ and GADPH levels were compared between each other and among treatments to yield a p value using ANOVA analysis.

## Results

### Growth and body composition

Treatment intervention applied was modest, thus differences in growth characteristics such as body weight were not expected and were not observed (Table 1). Differences among treatments were also not observed for body composition factors (Table 2),

**Table 1:** Growth factors of 23 wk old castrated pigs in control and feed withdrawal (FW) treatments.

		Treatments		Probability
		
Factor	Control	FWx1	FWx4	value
n	9	9	9	
Initial weight (Wt, kg)	13.7 ± 0.8	13.4 ± 0.4	14.6 ± 0.6	0.4057
Wt at 28 d kg	34.0 ± 1.8	36.0 ± 1.1	36.5 ± 1.1	0.4170
Wt at 56 d kg	63.7 ± 2.5	66.9 ± 1.8	67.6 ± 2.1	0.4037
Wt at 84 d kg	96.6 ± 2.8	100.3 ± 2.0	101.2 ± 2.9	0.4202
Wt at 124 d kg	135.8 ± 3.1	138.6 ± 1.9	140.6 ± 3.2	0.4122
Total wt gain kg	122.0 ± 2.6	125.0 ± 1.7	126.4 ± 3.0	0.4476
ADG kg	1.02 ± 0.02	1.05 ± 0.02	1.06 ± 0.02	0.4785
Total feed intake kg	339.2 ± 10.5	346.1 ± 8.3	358.3 ± 12.1	0.4355
Dry feed intake kg/d	2.8 ± 0.08	2.9 ± 0.07	3.0 ± 0.09	0.4095
Gain: feed	0.36 ± 0.01	0.36 ± 0.01	0.36 ± 0.01	0.7698

Control (no FW),

Single feed withdrawal (FWx1) for 48 h at 19 wk of age

Multiple feed withdrawal (FWx4) for 24 h at age 7 and 11 wk and for 48 h at 15 and 19 wk treatments

Values are expressed as mean ± SEM.

ADG Average daily gain

**Table 2:** Body composition characteristics of 23 wk old castrated pigs in control and feed withdrawal (FW) treatments.

		Treatments		Probability
		
Factor	Control	FWx1	FWx4	value
n	9	9	9	
Slaughter weight (Wt), kg	133.8 ± 3.0	136.3 ± 1.9	138.7 ± 3.4	0.4861
Hot carcass Wt, kg	111.2 ± 2.2	113.4 ± 1.6	115.2 ± 2.8	0.4434
Kidney fat (kg)	3.1 ± 0.2	2.9 ± 0.2	3.1 ± 0.2	0.7021
Carcass fat depth (mm)	24.0 ± 0.5	26.3 ± 1.1	26.9 ± 1.2	0.1238
Carcass lean depth (mm)	56.0 ± 1.0	58.6 ± 1.2	58.6 ± 1.6	0.2807
Lean yield estimate (%)	58.0 ± 0.2	57.5 ± 0.4	57.3 ± 0.4	0.2404
Yield/100 kg slaughter, Wt	94.4 ± 5.8	86.9 ± 7.5	74.3 ± 8.4	0.1668
Molecular marker; Muscle				
PPARγ - GADPH (% of control)	100 ± 150ab	267 ± 154a	57 ± 153b	0.0516
Total adipocyte number in muscle				
Cells/mg of muscle fat	860 ± 204.9	890 ± 9.0	754 ± 144.7	0.8339
Cells/mg of muscle	663 ± 158	748 ± 140.1	560 ± 135.0	0.6607

Control (no FW),

Single feed withdrawal (FWx1) for 48 h at 19 wk of age

Multiple feed withdrawal (FWx4) for 24 h at age 7 and 11 wk and for 48 h at 15 and 19 wk treatments

Values are expressed as mean ± SEM.

Carcass fat and lean depth - Destron system [37]

The fat depth measurement location is between the 3^rd ^and 4^th ^last ribs, 7.0 cm over from the midline.

The instrument used for grading was the Anitech PG-100 grading probe (Anitech Information System Inc., Markham, Ontario).

The % estimated lean yield is based on a quadratic equation which is also used as a reference for slaughter plants:

(68.1863-0.7833(F) + 0.0689(M) + 0.0080(F^2^) - 0.0002(M^2^) + 0.0006(FM). Where F is fat thickness in mm and M is muscle thickness in mm.

### Glucose tolerance test

**Figure 2:**
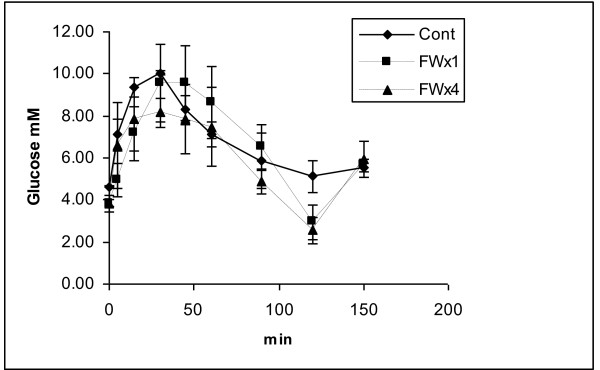
**Blood glucose concentration curves during the glucose tolerance test of castrated pigs at 21 wk of age undergoing control (no feed withdrawal), single feed withdrawal (FWx1) for 48 h at 19 wk or multiple feed withdrawal (FWx4), FW for 24 h at age 7 and 11 wk and for 48 h FW at 15 and 19 wk**. Trends (p = .1048 and .0968) towards decreased glucose concentrations were observed for fasting and 120 min post glucose consumption samples respectively from pigs in the FW treatments relative to values for pigs in the control treatment (n = 3).

**Figure 3:**
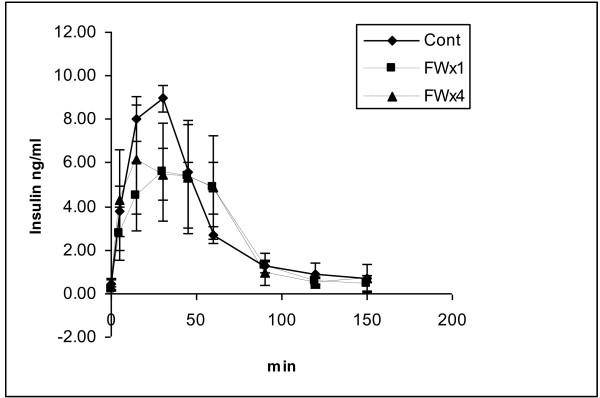
**Plasma insulin concentration curves during the glucose tolerance test of castrated pigs at 21 wk of age undergoing control (no feed withdrawal), single feed withdrawal (FWx1) for 48 h at 19 wk or multiple feed withdrawal (FWx4) for 24 h at age 7 and 11 wk and for 48 h at 15 and 19 wk**. (n = 3)

The blood glucose and insulin concentrations during the GTT are provided in Figures 2 and 3, respectively. Differences due to the FW treatment were not significant at any sampling time for either glucose or insulin. Only trends (p = 0.1048 and 0.0968) towards lower values were observed for blood glucose concentrations for samples collected prior to and 120 min post glucose meal consumption for samples collected from pigs in the FWx4 treatment. Despite these differences in blood glucose concentration, differences were not significant for glucose and insulin concentration, HOMA values or glucose and insulin AUC (Table 3).

**Table 3:** Glucose tolerance test factors and area under the curve (AUC_ground_) for glucose and insulin during the glucose tolerance test conducted in 21 wk old castrated pigs in control and feed withdrawal (FW) treatments.

		Treatments		Probability
		
Factors	Control	FWx1	FWx4	value
n	3	3	3	
Fasting glucose mM/L	4.64 ± 0.03	3.83 ± 0.41	3.85 ± 0.15	0.1048
Fasting Insulin μU/mL	11.02 ± 6.39	4.88 ± 2.51	7.65 ± 7.66	0.7716
HOMA index	2.28 ± 1.32	0.82 ± 0.41	1.26 ± 1.26	0.4844
AUC glucose (mM*min)	870.8 ± 37.1	851.7 ± 80.1	750.4 ± 57.1	0.3815
AUC insulin (ng/ml*min)	469.1 ± 42.5	429.8 ± 96.5	421.3 ± 95.8	0.9099
Omental tissue				
Molecular marker				
PPARγ - GADPH(% of control)	100 ± 23	105 ± 29	126 ± 13	0.1599
Total cell number/mg	1514 ± 171.1	1184 ± 112.6	1257 ± 114.8	0.3911

Control (no FW),

Single feed withdrawal (FWx1) for 48 h at 19 wk of age

Multiple feed withdrawal (FWx4) for 24 h at age 7 and 11 wk and for 48 h at 15 and 19 wk treatments

HOMA - Homeostatis model assessment index - [fasting insulin (μU/mL) * fasting glucose (mM/L)]/22.5

### Correlation coefficients among GTT factors

The AUC glucose was significantly related to AUC insulin, blood glucose concentrations at 30, 45 and 60 min and plasma insulin concentrations at 45 and 90 min post glucose meal consumption (Table 4). The AUC for insulin was in turn correlated to AUC for glucose and blood glucose and plasma insulin concentrations at 30, 45 and 60 min post the glucose meal consumption, suggesting that blood glucose concentration 45 min post glucose meal in a GTT was valuable in assessing the AUC for both glucose and insulin. The AUC for insulin was also correlated to the plasma insulin concentrations prior to glucose meal consumption.

**Table 4:** Correlation coefficients for parameters associated with area under the curve (AUC) for glucose and insulin during the glucose tolerance test conducted in 21 wk old castrated pigs from control and feed withdrawal (FW) treatments.

Parameter	Factor	Correlation coefficient	P value
AUC Glucose	AUC Insulin	0.6847	0.0419
	insulin at 45 min	0.7269	0.0265
	insulin at 90 min	0.6711	0.0478
	Glucose at 30 min	0.8432	0.043
	Glucose at 45 min	0.8504	0.0037
	Glucose at 60 min	0.7736	0.0145
			
AUC Insulin	AUC Glucose	0.6846	0.0419
	Insulin prior to glucose meal	0.7152	0.0303
	insulin at 30 min	0.8357	0.005
	insulin at 45 min	0.9345	0.002
	insulin at 60 min	0.6733	0.0468
	Glucose at 30 min	0.807	0.0086
	Glucose at 45 min	0.7063	0.0335
	Glucose at 60 min	0.6805	0.0437

Control (no FW),

Single feed withdrawal (FWx1) for 48 h at 19 wk of age

Multiple feed withdrawal (FWx4) for 24 h at age 7 and 11 wk and for 48 h at 15 and 19 wk treatments

n = 9

### Adipose tissue cellularity and glucose incorporation into fat

**Figure 4:**
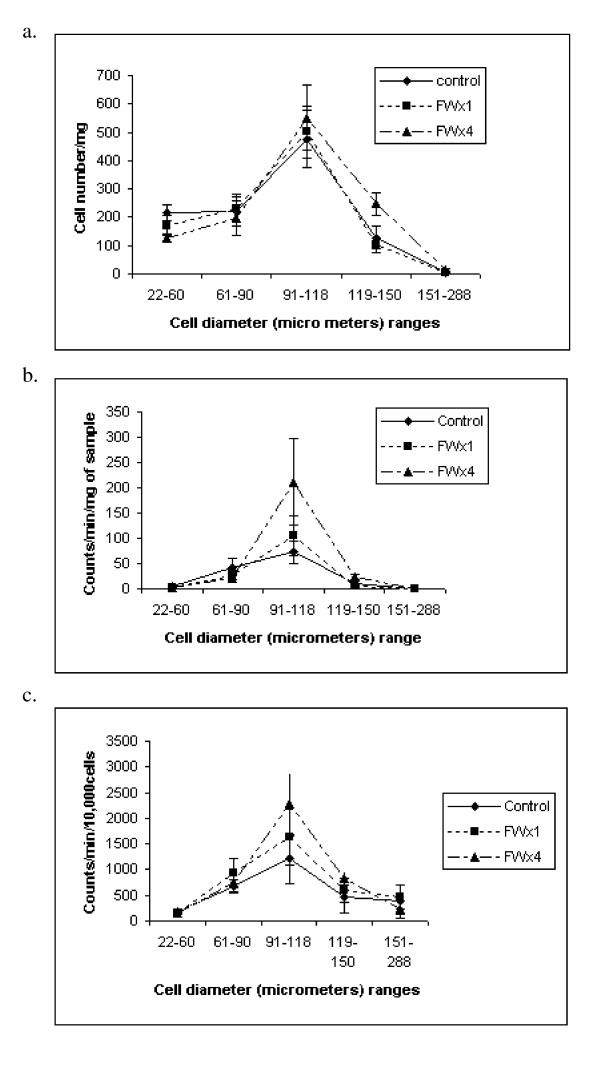
**Glucose Universal ^14^C uptake by cells of different diameter (μm) and from omental adipose tissue of pigs in the feed withdrawal (FW) treatments: control (no feed withdrawal), single FW (FWx1) for 48 h at 19 wk or multiple FW (FWx4) for 24 h at age 7 and 11 wk and for 48 h at 15 and 19 wk**. a. cells per/mg of fat sample, b. counts/min/mg of fat and c. counts/min/10^4 ^cells. Cell numbers and U^14^C glucose incorporation rate for cells in the diameter range of 119-150 μm tended (p = .0685 and .0872, respectively) to be greater for pigs in the FWx4 treatment relative to the other treatments, while counts per minute of fat/mg of sample was greater (p = .0234) for the same cells. (n = 5, 6 and 7 for control, FWx1, and FWx4 respectively)

In the OM adipose tissue differences among treatments were not observed (p > 0.05) for cell numbers in any of the diameter ranges studied, due to large variances, which may be due to the innate variation among the pigs, minimal replication and the extensive handling of the samples. As is evident in Figure 4a, the largest proportion of cells was in the 91-118 μm diameter range, but differences due to FW treatment were not significant. However, pigs in the FWx4 treatment had 191% greater (p = 0.0685) number of cells in the diameter range of 119-150 μm than the cells in pigs in the control treatment.

No difference in glucose incorporation into fat, measured as CPM from ^14^C from glucose U ^14^C incorporation into fat from the cells was noted for FW treatments, except for cells in the range of 119-150 μm (Figure 4b), where the CPM of fat from 1.0 mg of sample was 223% greater (p = 0.0234) than that of cells from pigs in the control or FWx1 treatment. The highest CPM for fat from cells/mg of sample was noted for cells in the 91-118 μm diameter range, but differences due to FW treatment were not significant. Similarly the rate of glucose uptake as CPM/10^4 ^cells was greatest for cells in the diameter range of 91-118 μm (Figure 4c) but treatment effects were obscured due the variation. However, the rate of glucose uptake for the cells in the 119-150 μm diameter range, from the pigs in the FWx4 treatment tended (p = 0.0872) to be higher than that of the pigs in either the no FW (control) or the FWx1 treatments by 173 and 139%, respectively.

**Figure 5:**
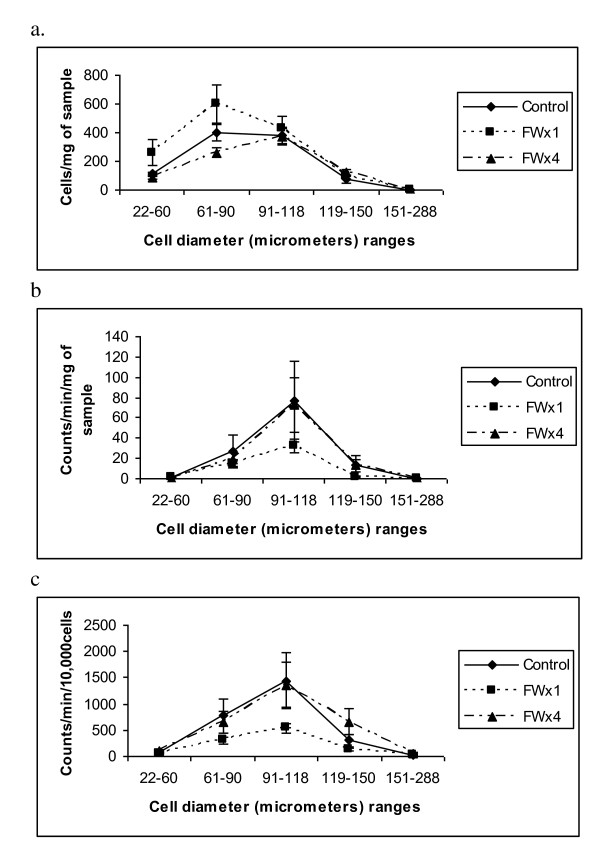
**Glucose Universal ^14^C uptake by cells of different diameter (μm) and from subcutaneous adipose tissue of pigs in the feed withdrawal (FW) treatments: control (no feed withdrawal), single FW (FWx1) for 48 h at 19 wk or multiple FW (FWx4) for 24 h at age 7 and 11 wk and for 48 h at 15 and 19 wk**. a. cells per/mg of fat sample, b. counts/min/mg of fat and c. counts/min/10^4 ^cells. Cell numbers in the diameter ranges of 22-60 and 60-90 μm tended (p = .0773, .0613 respectively) to be greater for pigs in the FWx1 relative to those in the FWx4 treatment. (n = 7, 6 and 7 for control, FWx1, and FWx4 respectively)

In the SQ tissue (Figure 5 a. b. c) the FW treatments tended (p = 0.0773 and 0.0613) to affect cell number in the diameter ranges of 22-60 and 60-90 μm, where the pigs in the FWx1 had 305 and 233% greater number of cells, respectively than those in the FWx4 treatment. Unlike that observed for the OM tissue, the cell number appeared to be distributed more evenly among the diameter ranges of 60-118 μm. Glucose uptake as CPM of fat/mg of sample or rate of glucose incorporation were not affected by treatment.

### PPARγ expression

The molecular marker PPARγ expression was determined with respect to the house keeping gene GADPH, to substantiate observations from the cellularity determinations. Although differences in total cell count from muscle were not affected by the FW treatment (Table 2) there was increased expression of PPARγ in muscle of pigs that underwent the FWx1 relative to those that underwent the FWx4 treatment but not relative to that in pigs in the control treatment. No differences in marker expression or cells/mg tissue in OM fat were observed in the present work (Figure 3a; Table 3). The PPARγ expression in SQ tissue was not undertaken and it was the only location where a trend towards an increase in cell numbers was observed for tissues obtained from pigs in the FWx1 treatment. However, this increase was noted only for cells with the diameter ranges of 20-60 and 60-90 μm and not for total cells/mg.

### Growth hormone concentrations

The FW treatment resulted in only relatively elevated values for porcine growth hormone of 3.26 ± 1.49 ng/mL while the value was 1.74 ± 0.74 ng/mL for the pigs from the control treatment indicating that growth hormone had no major involvement in altering the cell number and mirrored the effect observed for the molecular marker for adipocytes.

## Discussion

The lack of effect of the FW regimen on the growth characteristics of pigs was expected and is similar to that observed in rats [14] and cattle [10]. Intramuscular fat in heifers was increased in those subjected to FWx1 as evidenced by the increase in the percentage of heifers grading at the elevated grade. The aim of the present investigation was to determine if FW regimens could lead to an expansion in adipocyte number to improve glucose tolerance in the pig model by alleviating lipodystrophy and to assess application of FW regimens in humans to control progression of metabolic syndrome symptoms.

The FW duration (48 h) was similar to that applied in cattle [10] to insure complete absence of feed in the gastrointestinal tract, but when the pigs were 7 and 11 wk of age the duration of FW was 24 h as in rats [14] to minimize stress in the young animals. Although Liu et al. [27] imposed a 24 h fast prior to euthanasia in young pigs of comparable age to study the expression of adiponectin receptors because of their association to glucose metabolism, they did not study the effects of fasting on factors that affect glucose metabolism.

In the present study blood glucose appeared to be responsive to FW in a manner observed in the rats [14], where only blood glucose concentrations prior to glucose meal and at the 120 min post glucose meal were decreased by FWx4 regimen, but the HOMA and AUC was not affected by treatment. The fact that pre-glucose-meal values did not indicate a substantial or significant association with AUC for glucose (Table 4) is important as this concentration is often used to ascertain metabolic syndrome status in humans [15]. However the blood glucose concentrations at 45 min post the glucose meal indicated r values of 0.85 to AUC for glucose, furthermore this value was also highly associated with AUC for insulin. The values in the present study were derived with only nine pigs and would be worthy of validation because the type-2 diabetes status with respect to peak glucose and the AUC appear to influence coronary events and may be related to the higher incidence of coronary events in type2-diabetics [15].

Although the associations among glucose tolerance parameters and cellular factors could not be evaluated as in He et al. [14], it is evident from the Figure 4 and 5 that only cells ranging from 60 to 150 μm in diameter in the OM and SQ tissue had increased rates (CPM/10^4 ^cells) of glucose incorporation into fat relative to cells with smaller or larger diameters, upholding the hypothesis that cells of median diameter were most active in lipid synthesis and had the elevated lipogenic potential to participate in abating metabolic syndrome symptoms. In concurrence with Etherton et al. [4], differences in lipogenic activity were not evidenced in cells with diameter ranges between 60 to 150 μm. However in the present investigation, three diameter ranges within cell diameters from 60 to 150 μm were studied, while previously [4], only two cells size groups were considered within the same diameter range, thus they were unable to observe the peak in lipogenic activity in cells in the diameter range of 90 to 118 μm.

The cells from the pigs in the FWx4 treatment demonstrated elevated glucose uptake when determined as either CPM of fat from cells/mg of sample or as the CPM/10^4 ^cells and may be comparable to the observations of Fried et al. [12,28], where lipoprotein lipase activity was elevated in rats that weighed 300 g and were fasted for 72 h. Co-incidentally pigs in the FWx4 treatment had the lowest AUC for glucose. Although, it has been cited [29] that periodic fasting as a means of extending the lives of diabetics was attempted in the early part of the last century, with marginal success it has never been scientifically evaluated. The present study indicates that the adipose tissue expandability [17] especially of the OM [1] required to abate metabolic syndrome symptoms resides in the cells in the diameter range of 90 to 118 μm and maintenance of this expandability perhaps is possible through regular and periodic FW for 24 to 48 h.

In pigs the effect of periodic FW on the AUC for glucose appears to be independent of cell number and different from previous observations in rats [14], but the lack cellular response is concomitant with the non-significant response to cell markers investigated in the study for OM fat.

It is known that GH is a trophic hormone (through Insulin like Growth Factor) and that feed restriction [30] in cattle and fasting in humans [31] increases its concentration to act as a repartitioning agent, and to increase organomegaly [32,33]. Thus in sub-experiment 2 we evaluated the role of this hormone in adipogenesis. However despite the consistent elevation of the hormone in circulation in pigs euthanized post FW for 48 h, the difference was not significant and may point towards the lack of cellular response to FW in the pigs. However, the observed result perhaps represents the lack of sequential blood sampling and further definition of GH baseline, amplitude, mean overall and GH spike frequency. Further studies are required to examine GH secretory variables in more detail in response to FW.

It is of interest that concurrent changes occurred in AUC for glucose and lipogenic activity in the OM tissue, which has been implicated in precipitation of metabolic syndrome symptoms and progressively to type-2 diabetes in humans [34]. The relationship among adipocyte size, lipogenic activity and AUC of the glucose curve was initially highlighted by He et al. [14]. Since these associations warranted further investigation, especially, because it has been observed that adipose tissue from insulin sensitive adults had greater lipogenic enzyme activity than those from insulin resistant adults [5], the present study was conducted with pigs to demonstrate associations among FW regimen, glucose tolerance and glucose incorporation into fat in cells of varying diameter ranges. It can be further hypothesized that periodic FW may produce comparable effects as severe calorie restriction [35] or bariatric surgery [36], where fasting glucose levels declined significantly in type 2 diabetics prior to significant weight loss and eventually leading to reversal of type-2-diabetes. Similarly periodic FW for a determined time may delay development of type-2 diabetes especially if prudence in food consumption and energy utilization is exercised between FW.

In conclusion the data supports the hypothesis that FW enhances lipogenic potential of adipocytes especially in the OM adipose tissue in pigs and can consistently lower fasting and 120 min post glucose meal blood glucose concentrations. The heightened lipogenic potential of cells from pigs in the FWx4 treatment resides in cells with diameter ranges of 91 to 118 μm and significantly in cells with the diameter range of 119-150 μm.

## Abbreviations

ADG: Average daily gain; AUC: Area under the curve; CPM: Counts per minute; FW: feed withdrawal; FWx1: single feed withdrawal; FWx4: multiple feed withdrawal; GH: Growth hormone; GTT: Glucose tolerance test; HOMA: Homeostatic model assessment; KRB: Krebs-Ringer bicarbonate; OM: Omental fat; PPARγ: Peroxisome proliferator activated receptorγ; SQ: Subcutaneous fat; [U ^14^C]: Universal ^14^Carbon

## Competing interests

The authors declare that no competing interests existed.

## Authors' contributions

**PSM: **originator, principle applicant for the funding and coordinator of the project. **MLH **lead and participated in the glucose U^14^C uptake section of the experiment. **GT **was the technical assistant who completed the celluarity and radioactivity measurement from the samples. **TE **was the statistician who performed all the data handling. **TM **was member of the project team. **SM **was the technical assistant who conducted the live animal experiment, the GTT and the hormone experiment. **AS **was the leader and local coordinator of the experimental activities. **JM **lead the molecular marker analyses section of the experiment. **PL **was head of the radioactivity lab and assisted in proper usage of the facility. **EO **and **MVD **are advisors and project members. The final version of the manuscript has been seen by all the authors.

## Authors' information

**P. S. Mir: **Senior Research Scientist Agriculture and Agri- Food Canada (AAFC), Lethbridge AB. **M. L. He: **Post Doctoral Research Associate with Dr. McAllister. **G. Travis **Technical Assistant AAFC, Lethbridge with Dr. Mir. **T. Entz: **Statistician AAFC. Lethbridge. **T. McAllister: **Principal Research Scientist AAFC, Lethbridge. **S. Marchand: **is the Technical Assistant working with Dr. Schaefer. **A. Schaefer: **Principal Research Scientist AAFC, Lacombe AB. **J. Meadus: **Research Scientist AAFC, Lacombe. **P. Lepage: **Research Associate and Head of the radioactivity lab at AAFC, Lacombe. **E. Okine: **Chair of the Department and Professor of Ruminant Nutrition, University of Alberta and **M. Dodson: **Professor of Animal Science at Washington State University.
